# Integrative medicine for managing the symptoms of lupus nephritis: A protocol for systematic review and meta-analysis

**DOI:** 10.1097/MD.0000000000010224

**Published:** 2018-03-30

**Authors:** Tae-Young Choi, Ji Hee Jun, Myeong Soo Lee

**Affiliations:** Clinical Research Division, Korea Institute of Oriental Medicine, Daejeon, Republic of Korea.

**Keywords:** complementary and alternative medicine, herbal medicine, integrative medicine, lupus nephritis, protocol, randomized controlled trials, systematic review

## Abstract

**Background:**

Integrative medicine is claimed to improve symptoms of lupus nephritis. No systematic reviews have been performed for the application of integrative medicine for lupus nephritis on patients with systemic lupus erythematosus (SLE). Thus, this review will aim to evaluate the current evidence on the efficacy of integrative medicine for the management of lupus nephritis in patients with SLE.

**Methods and analyses:**

The following electronic databases will be searched for studies published from their dates of inception February 2018: Medline, EMBASE and the Cochrane Central Register of Controlled Trials (CENTRAL), as well as 6 Korean medical databases (Korea Med, the Oriental Medicine Advanced Search Integrated System [OASIS], DBpia, the Korean Medical Database [KM base], the Research Information Service System [RISS], and the Korean Studies Information Services System [KISS]), and 1 Chinese medical database (the China National Knowledge Infrastructure [CNKI]). Study selection, data extraction, and assessment will be performed independently by 2 researchers. The risk of bias (ROB) will be assessed using the Cochrane ROB tool.

**Dissemination:**

This systematic review will be published in a peer-reviewed journal and disseminated both electronically and in print. The review will be updated to inform and guide healthcare practice and policy.

**Trial registration number:**

PROSPERO 2018 CRD42018085205

## 1 Introduction

### 1.1 Description of the condition

Systemic lupus erythematosus (SLE) is a chronic systemic autoimmune disease^[1]^ that may impact virtually any organ system but very often injures the kidney; it predominantly affects women in young and middle adulthood.^[2]^ Lupus nephritis is a common but severe systemic impairment caused by SLE and a major cause of morbidity and mortality in patients with SLE.^[3]^ Lupus nephritis occurs in about half of all people with SLE,^[4]^ leading to end-stage kidney disease (ESKD) in 10% of patients.^[5]^ Thus, it requires treatment, which often involves substantial healthcare resources with considerable costs.^[6]^ Moreover, the health-related quality of life of SLE patients appears to be significantly worse than for patients with some other common chronic diseases.^[7]^ Therefore, an integrative approach may be appropriate.

### 1.2 Description of the intervention

Integrative medicine is a type of medical care that combines conventional (standard) medical treatment with complementary and alternative (CAM) therapies that have been shown to be safe and to work.^[8]^ One of the main components of CAM, the system of traditional Eastern Asian medicine (TEAM) is one of the whole-system CAM approaches used in Asia.^[9]^ In this review, we will focus on herbal medicine combined with conventional medicine.

### 1.3 How the intervention might work

The conventional medicine treatments for lupus nephritis patients, immunosuppressive medications and glucocorticoids, are often accompanied by side-effects, such as fatigue, weight gain, osteoporosis, and cataracts.^[10]^ It is claimed that an increase in the use of CAM with SLE patients^[11,12]^ reduces these symptoms, by up to 50% in the United States.^[13]^ However, it is unclear whether the evidence is reliable.

### 1.4 Why a review of this intervention is important

Recently published systematic reviews have concluded that integrative medicine used for lupus nephritis may improve the clinical efficacy of treatment and adverse drug reactions.^[14–17]^ These reviews include trials published only in Chinese, however, and have various shortcomings, such as insufficient searches, lack of quality assessment, and incorrectly applied meta-analysis methods.

### 1.5 Objective

To assess the efficacy and safety of integrative medicine for lupus nephritis in patients with SLE as reported in randomized controlled trials (RCTs).

## 2 Methods

### 2.1 Study registration

This protocol has been registered on PROSPERO 2018 CRD 42018085205 (http://www.crd.york.ac.uk/PROSPERO/display_record.php?ID=CRD42018085205).

### 2.2 Criteria for studies to be reviewed

#### 2.2.1 Types of studies

We will include in the review the RCTs of regardless of publications languages testing integrative medicine for lupus nephritis as identified by our search. We will exclude quasi-randomized trials, non-randomized trials, observational studies, case reports, abstracts, and letters.

#### 2.2.2 Types of participants

The patients will be adults (18 years or older) with a lupus nephritis diagnosis as confirmed according to the American College of Rheumatology Classification criteria for SLE.^[18]^

#### 2.2.3 Types of interventions and controls

Integrative medicine is defined as bringing herbal medicines and conventional medicine approaches together in a coordinated way. This will be applied as the intervention in the treatment group, while conventional medicine alone will be adopted in the control group. Herbal medicines will be defined as comprising traditional herbal treatments (decoctions, granules, capsules, pills, etc.). Conventional medicine will be defined as comprising conventional drug treatments and standard care methods. Studies comparing 2 types of herbal medicines will be excluded from the review. We will exclude other types of CAM including acupuncture, cupping, and etc.

#### 2.2.4 Type of outcome measures

The primary outcomes to be evaluated will be response rate, 24-hour urine protein, serum creatinine (SCr), and erythrocyte sedimentation rate (ESR). We will use the response rate as primary outcomes either “improved” or “not improved” where appropriate to enable the estimation of the relative risk of the defined outcomes. For instance, the participants’ change after allocated intervention graded as “recovery,” “markedly effective,” “effective,” and “ineffective” will dichotomized into “improved” or “not improved” by combining full or partial recovery into “improved,” and the rest (“ineffective”) into “not improved.” The secondary outcomes will be adverse events and quality of life likely to be related to treatment.

### 2.3 Search methods for study identification

#### 2.3.1 Electronic searches

The following electronic databases will be searched for studies published from their dates of inception to February 2018: Medline, EMBASE and the Cochrane Central Register of Controlled Trials (CENTRAL), as well as six Korean medical databases (Korea Med, the Oriental Medicine Advanced Search Integrated System [OASIS], DBpia, the Korean Medical Database [KM base], the Research Information Service System [RISS], and the Korean Studies Information Services System [KISS]) and one Chinese database (the China National Knowledge Infrastructure [CNKI]). In addition, the reference lists of potentially eligible articles will be searched manually to identify further relevant papers. Hard copies of all articles will be obtained and read in full. Finally, we will also search an international database (at www.clinicaltrials.gov) for trial registrations to identify ongoing or recently completed trials.

#### 2.3.2 Search strategy

Our search strategy will be conducted using the Medical Subject Headings (MeSH) [“integrative medicine” OR “traditional Chinese medicine” OR “herbal medicine” OR “herbal decoction”] AND [“lupus nephritis”]. This will be done using English, Chinese, and Korean.

### 2.4 Data collection, extraction, and assessment

#### 2.4.1 Selection of studies

Two independent reviewers (TYC and JHJ) will independently screen the titles and abstracts for potentially relevant studies, perform the study selection and record their decisions according to predefined criteria. Another reviewer (MSL) will resolve any disagreements in the study selection. The study selection will be documented and summarized in a Preferred Reporting Items for Systematic Reviews and Meta-Analyses (PRISMA)-compliant flow chart (at www.prisma-statement.org).^[19]^

#### 2.4.2 Data extraction

All articles will be read by 2 independent reviewers (TYC and JHJ), who will extract data from the articles according to predefined criteria. The extracted data will include specific details about the author name(s), year of publication, sample size, age and sex of participants, intervention (regimen), control (regimen), main outcomes, and adverse effects. When reported data are insufficient or unclear, an author will contact the first author or corresponding authors by e-mail or telephone to request missing or clarifying data.

#### 2.4.3 Assessment of risk of bias

Risk of bias (ROB) assessment will be performed using the ROB assessment tool from the Cochrane Handbook for Systematic Reviews of Interventions.^[20]^ The following characteristics will be assessed: sequence generation, allocation concealment, patient and personnel blinding, assessor blinding, reporting drop-out or withdrawal, intention-to-treat analysis, and selective outcome report. Thence, we will make a judgment on the quality of each trial according to the categories of “low,” “unclear,” and “high” ROB. We will categorize the selective outcome reporting bias as “low” only when the protocol of the study is available; otherwise, we will rate the ROB as “unclear” or “high.” ROB assessment for the included studies will be summarized in a table, and the results and implications will be critically discussed. Disagreements will be resolved by discussion among all the authors.

### 2.5 Data analysis

All statistical analyses will be conducted using the Review Manager (RevMan) software, version 5.3 (Cochrane Collaboration, Copenhagen, Denmark). Continuous data will be evaluated using the same instrument; the mean difference (MD) will be calculated, while the standardized mean difference (SMD) will be used for the same outcome but employing a different scale range. Dichotomous data will be assessed in terms of risk ratios (RR). Dichotomous and continuous variables will be expressed as efficacy values with 95% confidence intervals (CIs). If we detect heterogeneity (defined by results of tests of heterogeneity that indicate *P* < .1 by chi-square test and Higgins *I*^2^ ≥50%), subgroup analyses will be performed to find the cause of clinical heterogeneity. A random effects model will be used to assess combined effect sizes from efficacy variables; this will be done because substantial clinical heterogeneity is expected across the included studies based on the diversity of interventions, study design, and other conditions. If a sufficient number of included studies (at least 10 trials) are available, publication bias will be assessed using funnel plots and Egger regression method. Where sufficient data are available, we will conduct subgroup analyses for the primary outcomes to determine further, distinct evidence within the following subgroups.

We will use the Grading of Recommendations Assessment, Development and Evaluation (GRADE/GDT) web-based program (https://gradepro.org/) to determine the quality of evidence based on the Cochrane Handbook for Systematic Reviews of Interventions to create a Summary of Findings table.

### 2.6 Ethics and dissemination

Ethical approval is not required as this protocol is for a systematic review. The findings of this review will be disseminated widely through peer-reviewed publications and conference presentations.

## 3 Discussion

We start from the assumption that integrative medicine is effective for lupus nephritis in SLE patients. In this review, we will aim to identify the evidence that is currently available for this in order to better inform practice and guide future research and also gain useful information on acceptability to and applicability by clinicians for integrative treatments for managing SLE.

## Author contributions

**Conceptualization:** J.H. Jun, T-Y. Choi.

**Data curation:** J.H. Jun, T-Y. Choi.

**Funding acquisition:** M.S. Lee.

**Investigation:** M.S. Lee.

**Methodology:** T-Y. Choi.

**Supervision:** M.S. Lee.

**Writing – original draft:** M.S. Lee, T-Y. Choi.

**Writing – review & editing:** J.H. Jun.
